# Three-dimensional assessment of condylar head position in CBCT scans before and after orthognathic surgery

**DOI:** 10.1186/s12903-026-07720-0

**Published:** 2026-02-04

**Authors:** Kristina Schultze-Mosgau, Sebastian Gubik, Theresia Herterich, Andreas Vollmer, Niko Breitenbuecher, Hartmut Böhm, Alexander Kübler, Felix Kunz, Stefan Hartmann

**Affiliations:** 1 Department of Oral and Maxillofacial Plastic Surgery, University Hospital Würzburg, Würzburg, 97070 Germany; 2 Department of Orthodontics, University Hospital Würzburg, Würzburg, 97070 Germany

**Keywords:** Orthognathic surgery, Bilateral sagittal split osteotomy (BSSO), Condyle positioning, Condylar head positioning, Temporomandibular joint disorder, Computer-assisted surgery

## Abstract

**Background:**

Recent advances in orthognathic surgery—particularly the adoption of virtual 3D planning and patient-specific 3D-printed splints—have improved surgical precision, reduced operative time, and enhanced predictability of outcomes. Despite these developments, accurate intraoperative positioning of the mandibular condyle, especially the condylar head, during bilateral sagittal split osteotomy (BSSO), remains a major challenge. Although multiple techniques for condylar positioning have been proposed, their impact on postoperative condylar head position and joint space morphology has not been comprehensively assessed. This study aimed to evaluate postoperative changes in condylar head position and temporomandibular joint (TMJ) space using a Procrustes algorithm, and to investigate potential effects of surgeon handedness.

**Methods:**

A retrospective cone beam computed tomography (CBCT) analysis was conducted on 40 patients (24 female, 16 male) who underwent orthognathic surgery. The cohort comprised 27 patients with Angle Class II and 13 with Angle Class III malocclusion. Eighteen patients underwent bimaxillary procedures, and 22 underwent monomaxillary osteotomies. Condylar position and joint space dimensions were measured, and Procrustes shape analysis was applied to quantify TMJ space deformation. Statistical tests assessed positional changes and correlations with skeletal class, displacement, and surgeon handedness.

**Results:**

Significant postoperative changes were observed, with a bilateral reduction in cranial distances, reflecting a more caudal condylar head position, and a significant widening of the cranial joint space on the right side. Procrustes analysis confirmed measurable deformation of anterior and posterior TMJ compartments. Condylar heads were frequently positioned dorsally during initial registration, and results suggest that surgeon handedness may systematically influence condylar positioning.

**Conclusions:**

Postoperative condylar head position and TMJ space morphology are influenced by intraoperative handling, with surgeon handedness emerging as a potential contributor. Incorporating shape analysis methods such as Procrustes algorithms into future digital workflows may improve condylar positioning strategies and enhance surgical outcomes in BSSO.

## Introduction

Orthognathic surgery offers well-established techniques to correct skeletal discrepancies and dentofacial deformities, aiming to improve both function and facial aesthetics [1–3]. Since Hullihen’s first mandibular osteotomy in 1849, the field has witnessed substantial advancements. Bilateral sagittal split osteotomy (BSSO), initially described by Obwegeser and later refined by Dal Pont, has become a cornerstone procedure in mandibular correction [4, 5]. 

In parallel to surgical refinements, computer-assisted three-dimensional (3D) planning has markedly enhanced the precision, predictability, and reproducibility of orthognathic procedures [1, 6]. Current digital workflows allow for detailed preoperative planning of skeletal movements and occlusal outcomes. However, despite these technological advances, the accurate intraoperative and postoperative positioning of the mandibular condyle remains a persistent challenge.

Conventional splint-based planning systems focus primarily on the occlusion and skeletal relations of the jaws but often neglect the condylar head’s spatial relationship to the glenoid fossa. While various methods have been proposed to maintain or restore the preoperative condylar position [5, 6] intraoperative discrepancies frequently necessitate manual adjustments (e.g., condylar push-back) to achieve a satisfactory occlusion. This suggests that the condylar position defined by the preoperative CBCT alone may be insufficient for accurate transfer into surgical reality.

The present study aims to address this gap by analyzing the position of the condylar head throughout the orthognathic workflow—from initial registration and 3D planning to postoperative outcomes. By identifying potential parameters that influence condylar displacement, we seek to improve the integration of condylar positioning into future digital planning protocols.

## Materials and methods

### Patients

This study was conducted in accordance with the Declaration of Helsinki and approved by the Ethics Committee of the University of Würzburg (approval number: 2025-10-ka).

A total of 40 patients (24 female, 16 male) who underwent orthognathic surgery in the Department of Oral and Maxillofacial Plastic Surgery, University of Würzburg, between 2023 and 2024 were included. Selection criteria required that the entire mandibular fossa be visible on both pre- and postoperative cone beam computed tomography (CBCT) scans. The mean patient age was 30 years (range: 18–53 years).

### Methods

The cohort comprised 27 patients with Angle Class II and 13 with Angle Class III malocclusion. Eighteen patients underwent bimaxillary orthognathic surgery, and 22 underwent monomaxillary mandibular osteotomy. Preoperative scans were obtained with the occlusion in centric relation, defined using a wax bite. Postoperative occlusion was determined by the patient-specific surgical splint used intraoperatively. These splints were fabricated with IPS CaseDesigner^®^ (KLS Martin, Tuttlingen, Germany) to incorporate the virtually planned jaw displacements and rotations. All surgeries were performed by right-handed surgeons operating from the patient’s right side, ensuring consistent intraoperative access and potential laterality effects on condylar handling.

A) Methods to Analyze the Position of the Condylar Head

**Fig. 1:**
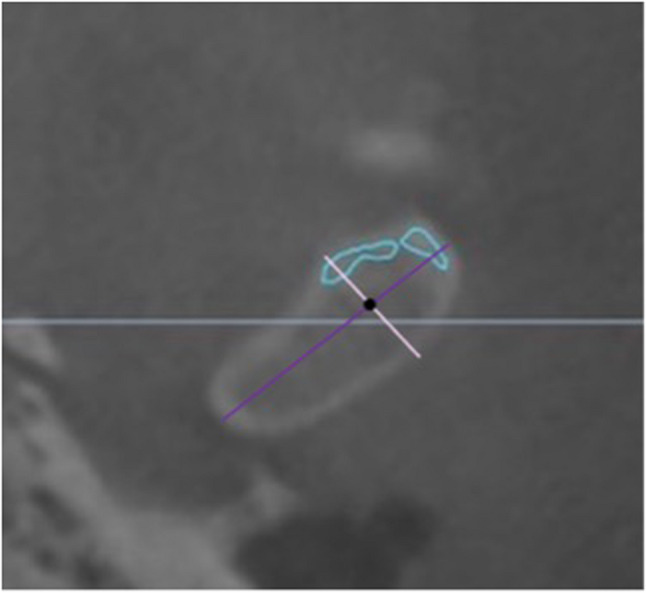
Determination of the center of the condyle in an axial slice of the CBCT

**Fig. 2:**
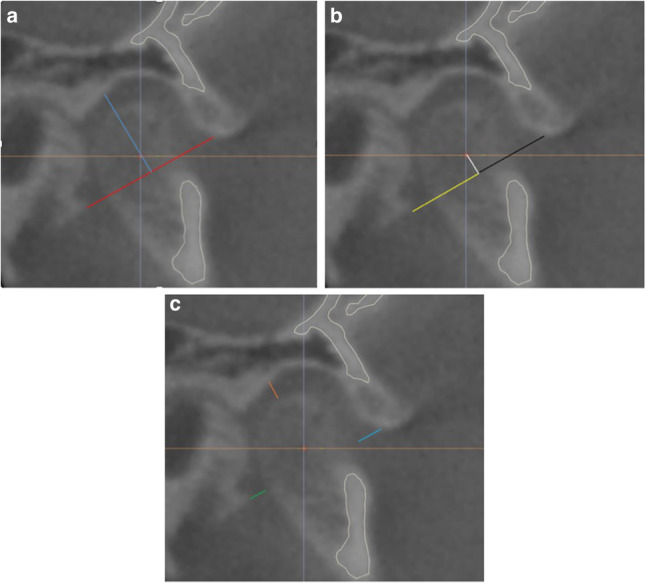
a,b,c: Measurement Reference Lines for Position Analysis

Pre- and postoperative CBCT scans were analyzed using IPS CaseDesigner^®^, which enables superimposition and direct comparison. Pre- and postoperative volumes were superimposed on the anterior cranial base to ensure homologous slice identification and anatomical correspondence before measurement. Within the software, multiplanar reconstruction of the CBCT volume ensured that slice selection and all coordinate definitions were consistently derived from the three-dimensional geometry of the skull and temporomandibular joint. Cephalometric landmarks were recorded according to commonly used parameters (e.g., A-point, B-point) but could be modified within the software to allow placement of custom points in specific CBCT regions. A custom algorithm was then applied to quantify condylar position: 

- Step 1 - Axial Slice(Appendix 1, Figure 1):
  - Identify the maximum medial–lateral dimension of the condylar head (dark purple line).
  - Identify the maximum anterior–posterior dimension in the same slice (light purple line).
  - The intersection of these lines defines the condylar center (black point).
- Step 2 – Sagittal Slice (Appendix 1, Figure 2 a, b, c):
  - Measure the greatest distance between the most ventral and dorsal caudal bony margins of the mandibular fossa (reference line, red).
  - Draw a perpendicular from the midpoint of the reference line to the cranial fossa roof (plumb line, blue).
  - Measure distances from the intersection of the reference and plumb lines to the ventral, cranial, and dorsal fossa margins, as well as to the condylar head center (black, white, and yellow lines, respectively).
  - Measure joint space widths in the ventral, cranial, and dorsal directions (turquoise, orange, and green lines).
  Although linear measurements were extracted from defined planes for visualization, all distances represent spatially referenced 3D metrics calculated from the registered volumetric dataset rather than planar 2D values.
- Step 3 – Cephalometric Analysis
  Conventional cephalometric analysis was performed, adding “Condylion left/right” as the most posterior–superior condylar point. This method differs from previous studies[7, 8], which referenced either the superior or inferior contour of the auditory meatus, by directly relating the mandibular fossa to the condyle for more accurate temporomandibular joint (TMJ) space analysis.
  Additional data extracted from IPS CaseDesigner® included:
  - Planned displacement distances (mm) for upper and lower jaws (sagittal right, anterior, left)
  - Upper jaw impaction (sagittal right, anterior, left)
  - Angle classification
  - Surgical technique (BSSO vs. bimaxillary osteotomy)

B) Analysis of TMJ Space Area

**Fig. 3:**
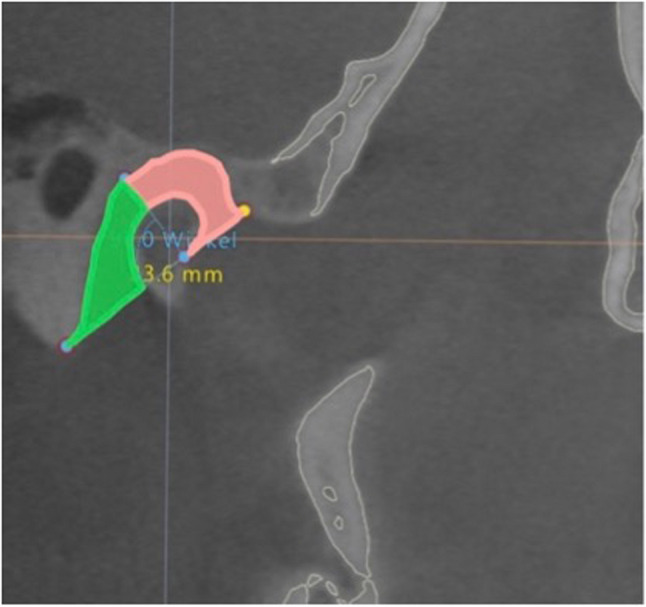
Label Studio - Example for Annotating the posterior and anterior area of the TMJ

The TMJ space was divided into anterior and posterior compartments using the line to the cranial fossa roof (Appendix 1, Figure 3). CBCT images were annotated in Label Studio (Heartex, San Francisco, CA, USA), and the areas were calculated for pre- and postoperative scans. Changes were quantified using the Procrustes algorithm.

C) Statistical Analysis:

Descriptive statistics (mean, median) and inferential tests (Shapiro–Wilk, paired t-test, independent samples t-test) were applied. Box plots were generated. Correlations between surgical displacement and condylar head movement were assessed, and correlation matrices were constructed.

Null Hypotheses (H0):

1. H0_1_: There is no significant difference between pre- and postoperative condylar head positions.
2. H0_2_: The extent of surgical displacement does not influence condylar head position.
3. H0_3_: The underlying skeletal anomaly does not affect condylar head position.

### Materials

CBCT analysis was performed in IPS CaseDesigner^®^ (KLS Martin, Tuttlingen, Germany). TMJ region annotations were conducted in Label Studio (Heartex, San Francisco, CA, USA). Statistical analyses were carried out in Python 3.12 (Python Software Foundation, Wilmington, DE, USA) using standard scientific libraries, with coding performed in Visual Studio Code 1.8 (Microsoft Corp., Redmond, WA, USA).

## Results

### Preoperative CBCT analysis

The dimensions of the condylar head and glenoid fossa were first assessed in the 40 patients. The mean lateral–medial width of the condylar head was 19.10 ± 2.74 mm on the left and 19.01 ± 2.85 mm on the right. The mean anterior–posterior width was 7.67 ± 1.35 mm on the left and 7.86 ± 1.20 mm on the right. Details of the glenoid fossa measurements are shown in Appendix 2, Table 1.

The preoperative position of the capitulum relative to the fossa was:

- Right side: ventral 8.20 ± 1.92 mm, dorsal 13.12 ± 3.41 mm, cranial 1.38 ± 2.12 mm.
- Left side: ventral 7.90 ± 1.68 mm, dorsal 13.28 ± 3.91 mm, cranial 1.15 ± 2.08 mm.

Mean preoperative joint space widths were:

- Right side: ventral 5.28 ± 2.28 mm, cranial 3.11 ± 1.30 mm, dorsal 7.48 ± 3.23 mm.
- Left side: ventral 5.35 ± 2.25 mm, cranial 3.32 ± 1.65 mm, dorsal 7.34 ± 3.99 mm.

**Fig. 4:**
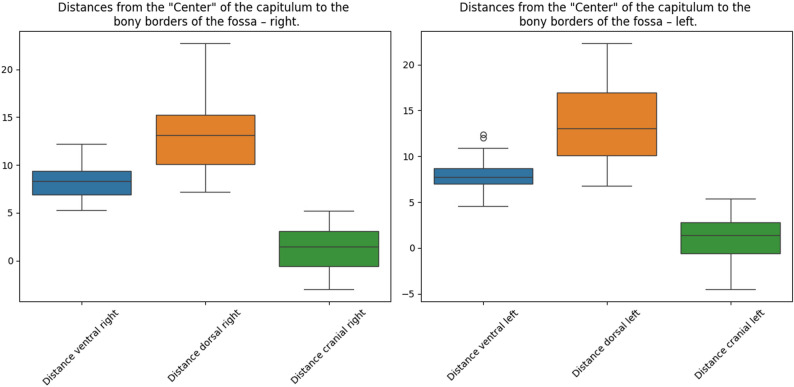
Preoperative Distances from the "Center" of the Capitulum to the bony borders of the fossa

**Fig. 5:**
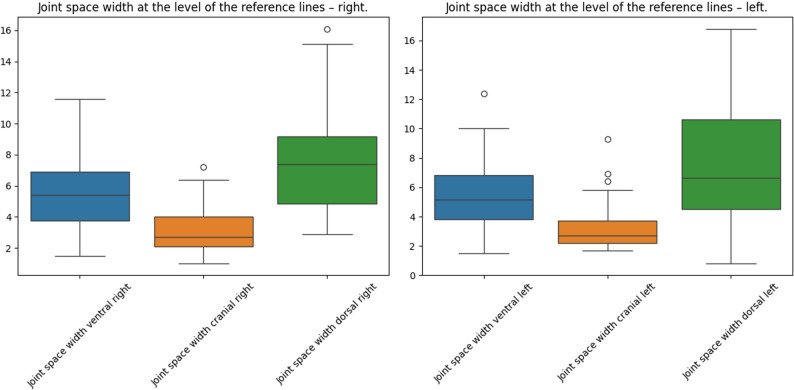
Preoperative Joint Space at the level of the reference lines

Detailed measurements are illustrated in Appendix 2, Figures 4–5.

### Postoperative CBCT analysis

**Fig. 6:**
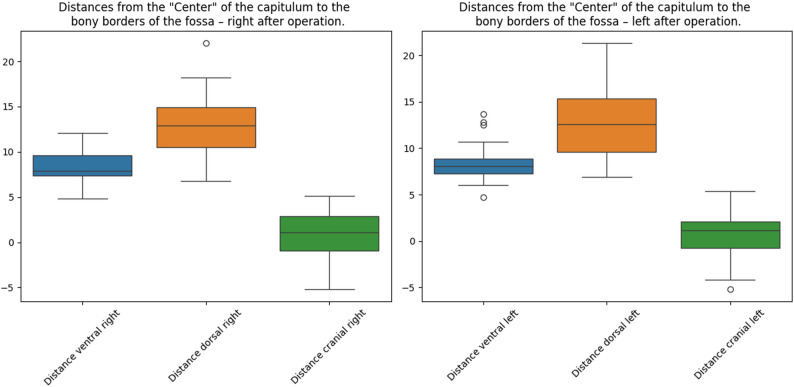
Postoperative Distances from the "Center" of the Capitulum to the bony borders of the fossa

**Fig. 7:**
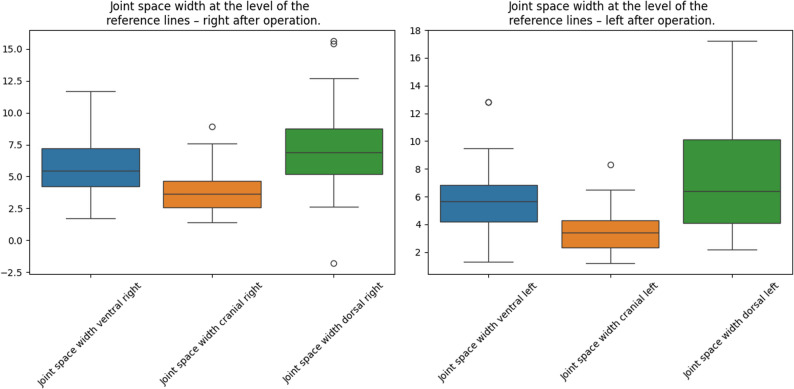
Postoperative Joint Space at the level of the reference lines

**Fig. 8:**
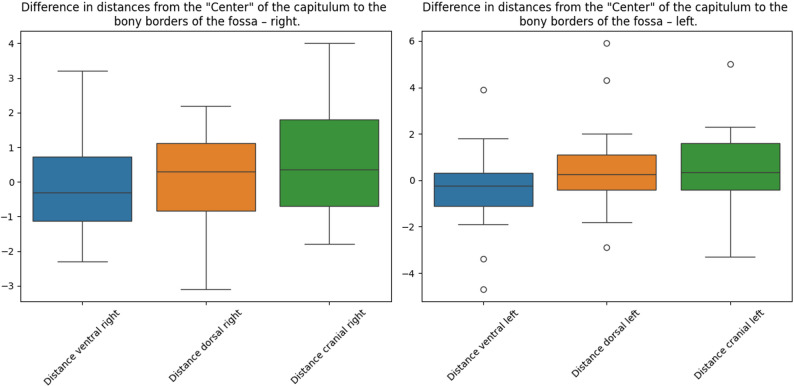
Pre- and Postoperative Difference of Distances from the "Center" of the Capitulum to the bony borders of the fossa

**Fig. 9:**
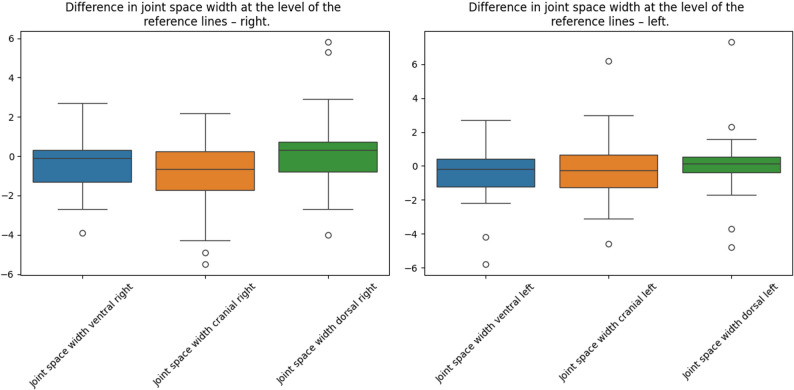
Pre- and Postoperative Difference of the Joint Space at the level of the reference lines

Postoperative capitulum positions relative to the fossa are shown in Appendix 2, Figures 6–7. Differences between pre- and postoperative measurements were analyzed using paired t-tests (Appendix 2, Table 2), with results visualized in Appendix 2, Figures 8–9. A significant bilateral reduction in cranial distance was observed, indicating a more caudal condylar head position, while the cranial joint space on the right side increased significantly (*p* = 0.011).

### Influence of skeletal class

Independent samples t-tests comparing Angle Class II and III cases revealed significant differences in cranial distance on the left side (*p* = 0.045) and cranial joint space width on the left side (*p* = 0.041) (Appendix 2, Table 3).

### Correlation with surgical displacement

**Fig. 10:**
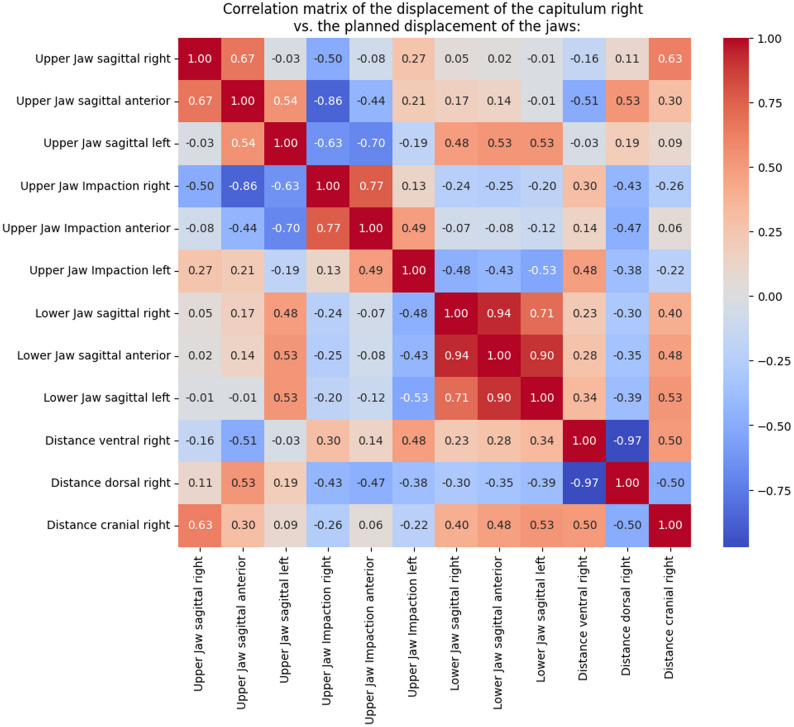
Correlation matrix of the displacement of the capitulum right vs. the planned displacement of the jaws

**Fig. 11:**
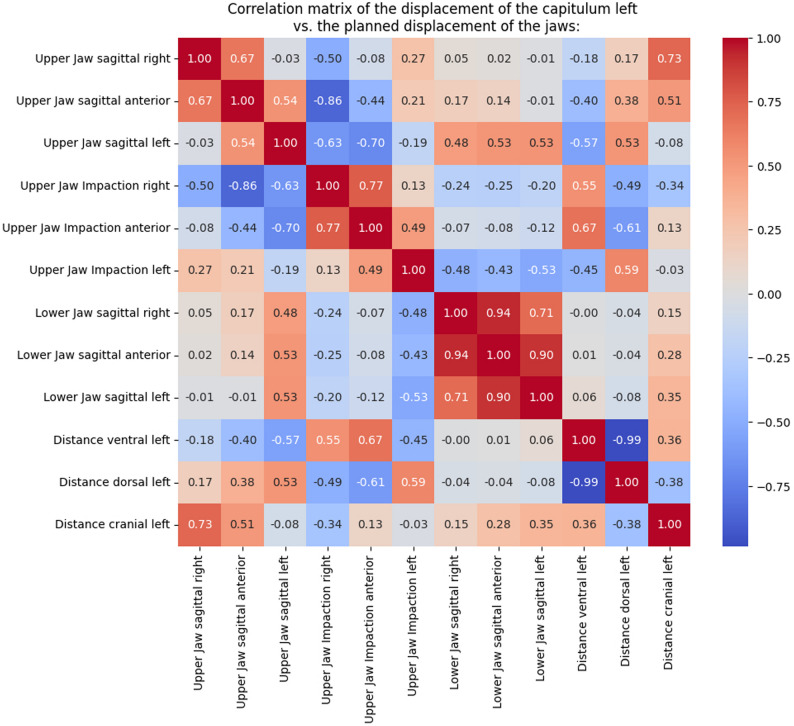
Correlation matrix of the displacement of the capitulum left vs. the planned displacement of the jaws

**Fig. 12:**
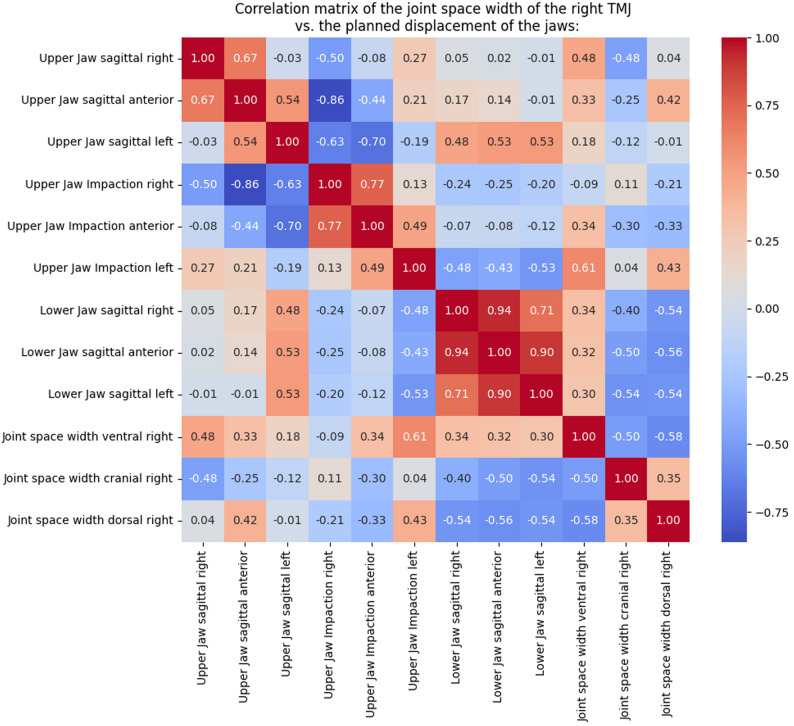
Correlation matrix of the joint space width of the right TMJ vs. the planned displacement of the jaws

**Fig. 13:**
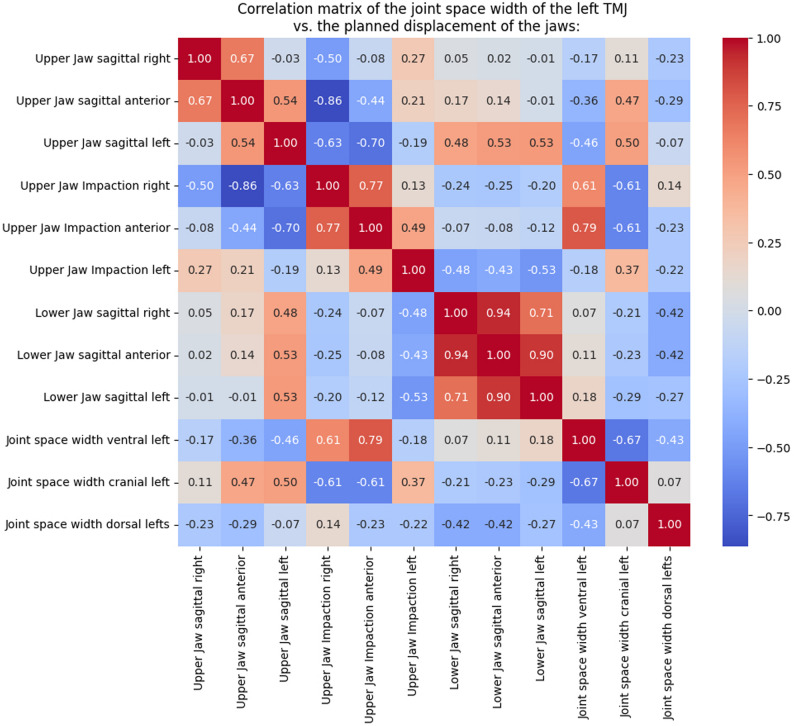
Correlation matrix of the joint space width of the left TMJ vs. the planned displacement of the jaws

Correlation matrices (Appendix 2, Figures 10–13) showed that greater mandibular advancement correlated positively with ventral and cranial distances, and negatively with dorsal distances.

### TMJ space area analysis

**Fig. 14:**
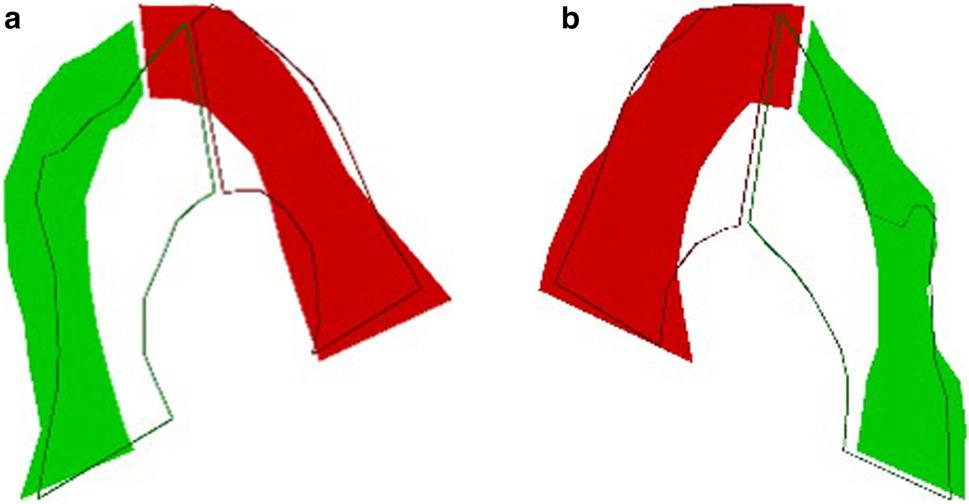
a, c: Deformation of the right (a) and left (b) temporomandibular joint areas. The preoperative regions are shaded, while the outlined areas represent the postoperative condition. Red indicates the anterior portion of the temporomandibular joint, and green represents the posterior portion

**Fig. 15:**
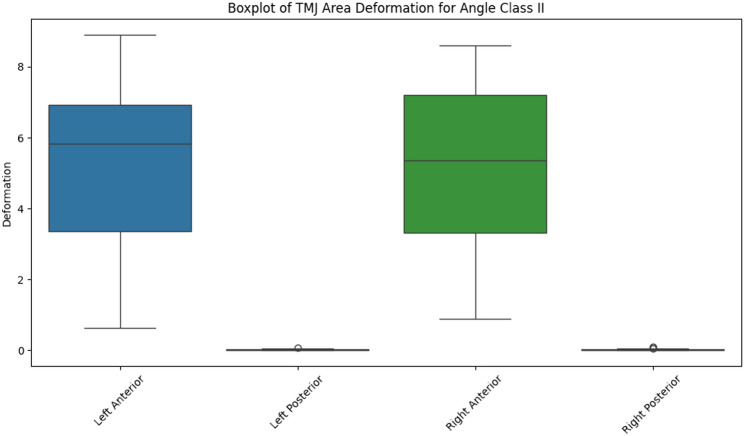
Boxplot Diagram of TMJ Area Deformation for Angle Class II

**Fig. 16:**
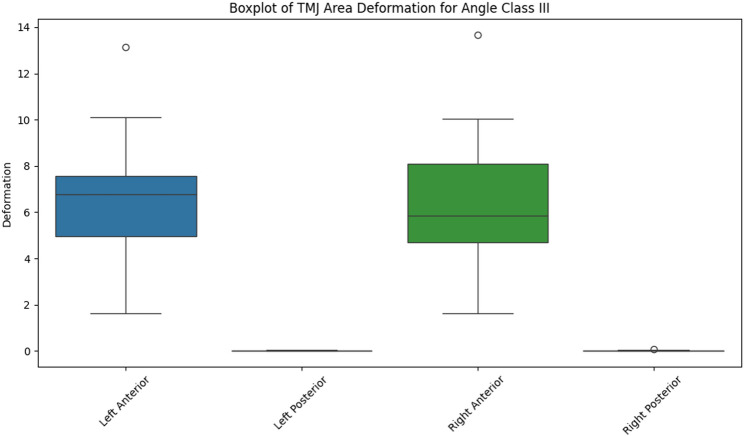
Boxplot Diagram of TMJ Area Deformation for Angle Class III

TMJ space area measurements for the left and right joints were analyzed using the Procrustes algorithm. An example of anterior and posterior TMJ area deformation in a single patient is shown in Appendix 2, Figure 14. Box plot diagrams for Angle Class II and III are presented in Appendix 2, Figures 15–16.

## Discussion

In orthognathic surgery, correct postoperative positioning of the condyle remains an important clinical objective, as suboptimal positioning has been linked to relapse and temporomandibular joint (TMJ) disorders [7–11]. While the surgeon can manipulate the condyle intraoperatively, the clinically relevant structure for analysis is the condylar head in relation to the glenoid fossa.

Preoperative CBCT analysis of the condylar head relative to the individual anatomical structures of the glenoid fossa can provide two types of information:

1. Potential warning signs of pre-existing TMJ disorders, such as arthrosis or cartilage damage.
2. Insights into the registration process before surgery, which is typically performed using a wax bite prior to CBCT acquisition.

In previous studies [12], joint space measurements in control groups demonstrated near-equal anterior and posterior spaces (e.g., anterior space: 2.12 ± 0.51/2.08 ± 0.47 mm; posterior space: 2.16 ± 0.44/2.09 ± 0.40 mm; superior space: 2.63 ± 0.39/2.71 ± 0.42 mm). In contrast, the present study found a consistently greater ventral than dorsal distance, suggesting that the condylar head is displaced dorsally during initial registration.

Statistical analysis confirmed significant changes in cranial distance on the right side (*p* = 0.019) and in cranial joint space width on the right side (*p* = 0.011). Postoperatively, cranial distances were reduced bilaterally, indicating a more caudal condylar position, while the cranial joint space widened on the right side. This pattern suggests a complex three-dimensional repositioning of the condyles rather than a simple intrusion–extrusion movement. The asymmetry, with caudal seating on both sides but joint space widening limited to the right, may reflect subtle mandibular rotation combined with the influence of surgeon handedness. All surgeons in this study were right-handed and operated from the patient’s right side, which likely contributed to the observed positioning. Comparable laterality effects have been reported in other surgical fields, including plastic and orthopedic procedures such as hip replacement and fracture fixation [11–13]. 

The cranial distances measured in this study were nearly twice those reported in comparable investigations [14], although differences in landmark definitions limit direct comparison. The observed tendency toward dorsal displacement is further supported by TMJ area deformation analysis, which consistently showed a thinner posterior than anterior compartment. Postoperative CBCT box plots for distances and joint spaces largely mirrored the preoperative distributions, with mean pre-to-postoperative changes in all directions remaining below 0.8 mm.

When stratified by skeletal class, significant differences were found between Angle Class II and III cases for cranial distance (left side, *p* = 0.045) and cranial joint space width (left side, *p* = 0.041). Correlation analysis further revealed that greater mandibular advancement was associated with increased ventral and cranial distances, while dorsal distances were negatively correlated.

Although the measured displacements were small - consistent with previous reports [15] - even minor condylar head shifts may contribute to TMJ dysfunction. Literature suggests that preoperative joint status [15] and postoperative disc position [10] may be more predictive of TMJ symptoms than minor bony positional changes. MRI studies could help clarify disc behavior post-surgery, though this is not routinely performed in orthognathic protocols. In long-term follow-up, Class III patients have been shown to exhibit condylar head repositioning toward baseline without compromising skeletal stability [16]. Importantly, the mean positional changes observed in our cohort remained below 0.8 mm, which lies within the clinically accepted threshold of < 1 mm for maintaining postpoperative TMJ stability. Despite the small magnitude of these displacements, even subtle condylar shifts can influence loading patterns within the joint and potentially contribute to pain, dysfunction, or relapse in susceptible individuals. Therefore, minimizing intraoperative condylar movement remains a critical objective during BSSO procedures.

Our results further support the concept that condylar behavior should be considered during preoperative planning, rather than only as an intraoperative adjustment variable. Integrating the condylar head’s starting position and predicted movement into virtual workflows may help reduce complication risk and improve postoperative TMJ function.

Additionally, the present data highlight the role of surgeon-related factors, such as handedness and operating position, in contributing to systematic condylar seating patterns. This emphasizes the importance of surgical awareness and technique-independent positioning aids to minimize operator-induced variability.

Because these seating tendencies were systematic — with dorsal positioning preoperatively and more caudal seating on the right side postoperatively — likely reflecting surgeon handedness, this finding has implications for virtual planning systems. Future research should incorporate normative data into CBCT-based positioning tools (e.g., IPS CaseDesigner^®^) to guide standardized condylar positioning and reduce reliance on surgeon technique.

## Conclusions

This study investigated the spatial positioning of the condylar head in patients undergoing orthognathic surgery using CBCT-based analysis. The findings indicate that condylar heads are frequently positioned dorsally during initial registration. Notably, a bilateral reduction in cranial condylar head distances was observed, reflecting a more caudal condylar head position, together with a significant widening of the cranial joint space on the right side. This supports the hypothesis that intraoperative handling and surgeon handedness systematically influence condylar head seating patterns. Because all surgeons involved were right-handed and operated from the patient’s right side, we hypothesize that laterality may influence intraoperative condylar head positioning.

Furthermore, the classification of skeletal malocclusion (Angle Class II vs. III) had no substantial or consistent impact on postoperative condylar displacement, with only two parameters—cranial distance and cranial joint space width on the left side—showing statistically significant differences. Only minor correlations were observed between the magnitude of jaw movement and condylar head position, suggesting that condylar shifts are influenced more by intraoperative handling and surgeon-related factors than by skeletal morphology or planned surgical vectors.

Even though the condylar head displacements measured were generally small and within clinically acceptable limits, preventing unnecessary movement of the condylar head remains essential to reduce the risk of postoperative TMJ dysfunction, relapse, and condylar remodeling. Based on our findings, condylar positioning should be consciously incorporated into virtual planning and surgical execution. Greater awareness of surgeon-dependent influences—such as handedness and intraoperative access—may help maintain a physiologic condylar position and thereby optimize functional outcomes after BSSO.

## Data Availability

The datasets generated and analyzed during the current study are available from the corresponding author on reasonable request.

## Appendix 2

B1. Tables

**Table:** Anatomical measurements of the condylar head and the glenoid fossa

	Count	Mean	SD	Min.	Max.
Condylar Head left lateral-medial	40.00	19.10	2.74	13.00	24.20
Condylar Head left anterior-posterior	40.00	7.67	1.35	4.40	11.10
Condylar Head right lateral-medial	40.00	19.01	2.85	11.90	22.70
Condylar Head right anterior-posterior	40.00	7.86	1.20	4.40	10.40
Fossa left ventral-dorsal	40.00	21.17	4.38	15.10	24.20
Fossa left cranial-caudal	40.00	9.78	1.52	6.80	11.10
Fossa right ventral-dorsal	40.00	21.35	3.71	15.50	22.70
Fossa right cranial-caudal	40.00	10.01	1.26	7.70	10.40

**Table:** Paired t-Test on the distances and joint spaces pre- and postoperative for the right and left condylar head

Comparison: Pre- and Postoperation	t-Test-Statistics	*p*-value
Distance ventral right	1.012	0.318
Distance dorsal right	−0.892	0.378
Distance cranial right	−2.438	0.019
Joint space width ventral right	1.953	0.058
Joint space width cranial right	2.673	0.011
Joint space width dorsal right	−0.668	0.508
Distance ventral left	1.723	0.093
Distance dorsal left	−1.896	0.065
Distance cranial left	−2.085	0.044
Joint space width ventral left	1.438	0.158
Joint space width cranial left	0.702	0.487
Joint space width dorsal left	−0.480	0.634

**Table:** Independent samples t-Test on the distances and joint spaces pre- and postoperative for the right and left condylar head comparing angle class II and III

Angle Class III vs. II	t-statistic	*p*-value
Distance ventral right	−0.325	0.747
Distance dorsal right	0.243	0.810
Distance cranial right	0.405	0.689
Joint space width ventral right	0.746	0.460
Joint space width cranial right	−0.093	0.927
Joint space width dorsal right	−0.977	0.336
Distance ventral left	0.842	0.409
Distance dorsal left	−1.041	0.306
Distance cranial left	2.109	0.045
Joint space width ventral left	1.914	0.063
Joint space width cranial left	−2.128	0.041
Joint space width dorsal left	−1.365	0.183
